# The wobbler mouse, an ALS animal model

**DOI:** 10.1007/s00438-013-0741-0

**Published:** 2013-03-29

**Authors:** Jakob Maximilian Moser, Paolo Bigini, Thomas Schmitt-John

**Affiliations:** 1 Molecular Biology and Genetics Department, Aarhus University, C. F. Møllers Alle 3, 8000 Aarhus C, Denmark; 2 Instituto di Ricerche Farmacologiche “Mario Negri” – IRCCS, Via La Masa 19, Milano, 20156 Italy

**Keywords:** ALS, Wobbler, Motor neuron degeneration, GARP complex, Vesicle tethering

## Abstract

This review article is focused on the research progress made utilizing the wobbler mouse as animal model for human motor neuron diseases, especially the amyotrophic lateral sclerosis (ALS). The wobbler mouse develops progressive degeneration of upper and lower motor neurons and shows striking similarities to ALS. The cellular effects of the *wobbler* mutation, cellular transport defects, neurofilament aggregation, neuronal hyperexcitability and neuroinflammation closely resemble human ALS. Now, 57 years after the first report on the wobbler mouse we summarize the progress made in understanding the disease mechanism and testing various therapeutic approaches and discuss the relevance of these advances for human ALS. The identification of the causative mutation linking the *wobbler* mutation to a vesicle transport factor and the research focussed on the cellular basis and the therapeutic treatment of the wobbler motor neuron degeneration has shed new light on the molecular pathology of the disease and might contribute to the understanding the complexity of ALS.

## Introduction

The wobbler mouse has successfully been used as animal model for human motor neuron diseases, especially ALS in the investigation of both, pathology and therapeutic intervention, as reviewed by (Boillee et al. 2003). In the recent years substantial progress has been made in understanding the molecular basis of the ALS-like wobbler motor neuron degeneration and testing therapeutic interventions. This article will briefly introduce the general progress in ALS research, will review the specific progress made on the understanding of molecular and cellular basis of the wobbler motor neuron disease and will provide an overview over therapeutic treatments tested in wobbler mice, which in itself have contributed to the understanding of the disease.

## Amyotrophic lateral sclerosis

Amyotrophic lateral sclerosis (ALS) was first described in 1869 by Jean-Martin Charcot and is the most common motor neuron disease (MND) among adults (Bruijn et al. 2004). Age of onset is typically between 50 and 60 years although earlier onset is not uncommon (Bruijn et al. 2004). Roughly 2 out of 100,000 individuals develop ALS and men have a slightly elevated risk compared to women (Ferraiuolo et al. 2011). 5–10 % of ALS cases can be attributed to familial forms (fALS), which predominantly are inherited in an autosomal dominant way. However, the vast majority of cases occur sporadically with no clear inheritance and thus, are termed sALS (Ferraiuolo et al. 2011). sALS is thought to be caused by a combination of genetic susceptibility and possible environmental factors (Ferraiuolo et al. 2011). Even with the emerging wider knowledge of the primary cause for at least a subset of ALS cases, the molecular and cellular pathomechanism of ALS is still poorly understood and was recently reviewed by (Ferraiuolo et al. 2011).

Clinically ALS can be described by loss of motor neurons at all levels of the motor system; from motor cortex to the ventral horn of the spinal cord (Mitchell and Borasio 2007). Previously, ALS was considered to be a disease, only affecting motor neurons, but recent evidence suggests the involvement of both sensory and spinocerebellar pathways and other neurons in the brain (Ferraiuolo et al. 2011). ALS is progressive and always fatal, leading to death within 3–5 years after onset of disease symptoms, resulting from failure of the respiratory muscles, being the most common cause of death (Bruijn et al. 2004; Wood-Allum and Shaw 2010). The loss of motor neurons leads to spasticity, hyperreflexia (upper motor neurons), generalized weakness, paralysis and muscle atrophy (lower motor neurons) (Bruijn et al. 2004). Pharmacological treatment options for ALS patients are very limited. Currently, only one FDA-approved drug, Riluzole, is available. The glutamate release inhibitor has a mild disease modifying effect and prolongs survival for about 3–4 months (Wood-Allum and Shaw 2010). Understanding the mechanisms underlying ALS and the effects preceding the onset of clinical symptoms is paramount in order to develop an effective treatment for ALS patients.

## Pathomechanisms of ALS

The identification of affected genes in some of the familial ALS cases (Table 1) and the generation and investigation of ALS animal models (Table 2) has shed some light on the causes of ALS pathology and the contributing factors. ALS has been linked to oxidative stress, excitotoxicity, abnormal aggregation of protein and defects of vesicle- and axonal transport systems as potential causes of the neurodegeneration, but also the neuroinflammatory processes are considered to contribute to the disease progression (Ferraiuolo et al. 2011).

**Table 1:** List of fALS genes and loci

OMIM #	fALS	Genomic location	Mode of inheritance	Gene/protein (symbol)	Cellular process	Reference
105400	ALS1	21q22.1	Dominant	Superoxide dismutase 1 (SOD1)	ROS detoxification	(Rosen 1993)
205100	ALS2	2p33	Recessive	Alsin (ALS2)	Vesicle traffic	(Yang et al. 2001)
606640	ALS3	18q21	Dominant	Unknown	Unknown	(Hand et al. 2002)
602433	ALS4	9q34	Dominant	Senataxin (SETX)	Transcription, RNA processing	(Chen et al. 2004)
602099	ALS5	15q15-21	Dom./Rec.	Unknown	Unknown	(Hentati et al. 1998)
608030	ALS6	16q11	Recessive	Fused in sarcoma (FUS)	DNA repair, transcription regulation	(Kwiatkowski et al. 2009)
608031	ALS7	20p13	Dominant	Unknown	Unknown	(Sapp et al. 2003)
608627	ALS8	20q13.3	Dominant	Vesicle-associated protein B (VAPB)	Vesicle Traffic	(Nishimura et al. 2004)
611895	ALS9	14q11.2	Dominant	Angiogenin (ANG)	RNase, angiogenesis	(Greenway et al. 2006)
612069	ALS10	1q36	Dominant	TAR DNA-binding protein (TARDP, TDP43)	DNA binding; transcription regulation and splicing	(Sreedharan et al. 2008)
612577	ALS11	6q21	Dominant	SAC domain-containing inositol phosphatase 3 (FIG4)	Lipid metabolism, endosome	(Chow et al. 2009)
613435	ALS12	10p13	Dom./rec.	Optineurin (OPTN)	RNA stabilization, autophagy?	(Maruyama et al. 2010)
601517	ALS13 (SCA2)	12q24.12	Dominant	Ataxin 2 (ATXN2)	Poly-glutamate stretches	(Pulst et al. 1996)
613954	ALS14	9p13.3	Dominant	Valosin-containing Protein (VCP)	ER protein export, autophagy	(Johnson et al. 2010)
300857	ALS15	Xp11.21	X-linked dom.	Ubiquilin 2, (UBQLN2)	Ubiquitin-like, protein degradation	(Deng et al. 2011)
614373	ALS16	9p13.3	Dominant	Sigma nonopioid intracellular receptor 1 (SIGMAR1)	Signalling receptor, ion channel regulation	(Al-Saif et al. 2011)
614696	ALS17	3p11.2	Dominant	Charged multivesicular body protein 2B (CHMP2B)	Vesicle traffic, endosomal sorting	(Parkinson et al. 2006)
614808	ALS18	17p13.2	Dominant	Profilin 1 (PFN1)	Actin filament assembly	(Wu et al. 2012)
105550 614260	FTD-ALS	9p21	Dominant	C9ORF72	Unknown function	(DeJesus-Hernandez et al. 2011; Renton et al. 2011)

**Table 2:** List of ALS animal models

Gene	Animal model, mutation	Species	Reference
SOD1 (ALS1)	Transgenic mice of 13 different SOD1 mutant variants are available.	Mouse	(Gurney et al. 1994), and others reviewed in (Bruijn et al. 2004)
	Transgenic SOD1 mutant variants	Zebrafish	(Lemmens et al. 2007; Ramesh et al. 2010)
Alsin (ALS2)	Alsin KO mouse	Mouse	(Devon et al. 2006)
	Alsin knock down	Zebrafish	(Gros-Louis et al. 2008)
FUS (ALS6)	Transgenic human WT FUS	Mouse	(Mitchell et al. 2013)
	Knock down and transgenic mutant FUS	Zebrafish	(Kabashi et al. 2010)
TARDP (TDP43, ALS10)	TARDP Knock out	Mouse	(Kraemer et al. 2010)
	Transgenic mutant TARDP	Mouse	(Wegorzewska et al. 2009)
	Transgenic mutant TARDP	Zebrafish	(Kabashi et al. 2010)
	Transgenic mutant TARDP	Rat	(Zhou et al. 2010)
Vps54^*wr*^	*Wobbler*, spontaneous recessive point mutation in vesicular/vacuolar protein sorting 54	Mouse	(Schmitt-John et al. 2005)
Tbce^*pmn*^	*pmn*, spontaneous recessive point mutation in the tubulin-specific chaperone-E	Mouse	(Bommel et al. 2002; Martin et al. 2002)
Nefl	Neurofilament light chain overexpressing transgenic mouse	Mouse	(Xu et al. 1993)
Nefh	Neurofilament heavy chain overexpressing transgenic mouse	Mouse	(Cote et al. 1993)
Prph	Peripherin overexpressing transgenic mouse	Mouse	(Beaulieu et al. 1999)

ALS has initially been linked to oxidative stress through mutations in the superoxide dismutase 1 (SOD1) gene (ALS1 locus), coding for a major antioxidant protein. SOD1 is an enzyme involved in the detoxification of free superoxide radicals and more than 100 different ALS-associated mutations have been described in the SOD1 gene so far (Ferraiuolo et al. 2011). Mutations are found both in fALS cases, where they constitute around 20 % of all cases, as well as in sALS cases (Mitchell and Borasio 2007). These mutations are dominantly inherited indicating that a gain of toxic function rather than loss-of-SOD1 function underlies the disease mechanism (Ferraiuolo et al. 2011). This is supported by the finding that abolishing the activity of the copper chaperone for SOD1, rendering SOD1 enzymatically inactive, no change in disease onset or progression was seen in SOD1 transgenic model mice (Subramaniam et al. 2002). However, markers for oxidative stress have been found in cerebrospinal fluid (CSF), serum and urine isolated from ALS patients (Smith et al. 1998; Simpson et al. 2004; Mitsumoto et al. 2008) and increased oxidative damage to proteins, DNA, lipids and mRNA species have been reported in tissue from both f- and sALS-patients (Shaw et al. 1995b; Fitzmaurice et al. 1996; Shibata et al. 2001; Chang et al. 2008). A further indication that oxidative stress plays a general role in motor neuron injury is supported by the findings that the fALS-associated TAR DNA-binding protein 43 (TDP-43; ALS10), a protein involved in the RNA machinery, is able to induce oxidative stress in a motor neuron-like cell line (Duan et al. 2010). Oxidative stress might be not a direct cause of ALS, but instead might aggravate other cellular effects of ALS, such as glutamate-induced excitotoxicity, mitochondrial impairment, protein aggregation, ER stress and signalling from neuronal support cells, thus aggravating motor neuron injury (Duffy et al. 2011; Wood et al. 2003; Kanekura et al. 2009; Blackburn et al. 2009; Sargsyan et al. 2005).

Mitochondrial dysfunction seems to be involved in at least some ALS cases. Mitochondria are involved in calcium homeostasis, intracellular energy production and control of apoptosis. SOD1-mutant mice display protein aggregates in the mitochondrial intermembrane space (Wong et al. 1995). Dysregulation of energy metabolism has been shown in both ALS patients and murine SOD1 models (Wiedemann et al. 2002; Mattiazzi et al. 2002). Likewise, altered morphology of mitochondria has been observed in skeletal muscle and spinal motor neurons in both ALS patients and in mouse models (Sasaki and Iwata 2007; Wong et al. 1995).

Excitotoxicity is thought to play a role in ALS. Glutamate is the main excitatory neurotransmitter in the central nervous system (CNS). Some ALS patients show increased levels of glutamate in the cerebrospinal fluid (CSF) (Shaw et al. 1995a). In affected areas of the CNS of sALS and fALS patients, decreased expression and reduced activity of the excitatory amino acid transporter 2 (EAAT2), an astroglial glutamate re-uptake transporter, was reported (Rothstein et al. 1992, 1995; Fray et al. 1998). The overexpression of EAAT2 (Guo et al. 2003) and the pharmacologically induced up-regulation of EAAT2 transcription could show beneficial effects in ALS model mice (Rothstein et al. 2005); suggesting that decreased EAAT2 expression is rather a cause than a consequence of ALS. Altered composition of the α-amino-3-hydroxy-5-methyl-4-isoxazolepropionic acid (AMPA) receptor in motor neurons of ALS patients could affect Ca^2+^ homeostasis and thus lead to neuronal death (Spalloni et al. 2004; Kwak et al. 2010). Altered expression of AMPA receptor subunits was also shown in mutant SOD1 transgenic ALS model mice and the treatment with an AMPA antagonist could ameliorate the disease progression in these mice (Tortarolo et al. 2006). Hyperexcitability of the motor system has been shown already in pre-symptomatic or early stages of human ALS cases (Vucic et al. 2008). The different aspects of glutamate-induced excitotoxicity, seen in ALS patients, can also be seen in mutant SOD1 transgenic mice (Meehan et al. 2010). In addition, it has been shown that mitochondria isolated from the CNS of SOD1 transgenic mice have altered calcium-buffering properties, which appears to have an effect on calcium-mediated excitotoxicity (Damiano et al. 2006).

Aggregation of proteins is a prominent feature of most neurodegenerative disorders. Accumulation of mis-folded or abnormal proteins into compact or skein-like ubiquitin-positive inclusions is a hallmark of ALS. TDP-43 has been shown to be a major constituent of these inclusions and is a common feature for both fALS and sALS (Neumann et al. 2006; Sreedharan et al. 2008). Normally, TDP-43 is predominantly present in the nucleus, but in the presence of TDP-43-positive inclusions, loss of nuclear TDP-43 is seen (Neumann et al. 2006). Redistribution of TDP-43 is an early event in at least some sALS cases, and TDP-43 mis-localisation and inclusion bodies are seen not only in motor neurons but also in the hippocampus of some patients (Giordana et al. 2010).

Patients suffering from SOD1-related ALS display inclusions containing SOD1, but not TDP-43 (Shibata et al. 1994; Tan et al. 2007). Likewise, cytoplasmic FUS (Fused in Sarcoma) inclusions are seen in patients with FUS-related ALS (ALS6) (Groen et al. 2010; Hewitt et al. 2010). Recently, mutations in Profilin 1 (PFN1), which is crucial for the conversion of g-actin to filamentous f-actin, have been linked to fALS (ALS18) with ubiquitinated insoluble inclusions including TDP-43 and mutant PFN1 (Wu et al. 2012). In some ALS cases, especially SOD1-related, neurofilament-rich hyaline conglomerate inclusions are seen in the perikaryon and the proximal dendrites (Ince et al. 1998) and increased neurofilament phosphorylation of was observed in these aggregates (Sobue et al. 1990). Altered stoichiometry and phosphorylation of neurofilament proteins could lead to impaired axonal transport and subsequent malfunctioning of axonal compartments (Ferraiuolo et al. 2011). Ubiquilin 2, a protein involved in the degradation of ubiquitinated proteins, has recently been shown to be part of inclusion bodies in ALS patients with various genetic causes. This suggests that the protein degradation pathway might play a more general role in ALS (Deng et al. 2011). Mis-folded proteins in the endoplasmic reticulum (ER) trigger the activation of the ER stress response pathway. The first step is recognition of mis-folded proteins by ER-resident chaperones, then the cells activate a collection of signal transduction pathways defined as the “unfolded protein response” (UPR), whose goal is to re-establish the cellular homeostasis (Kaufman 2002). In murine SOD1 models increased levels of UPR proteins precede the disease onset (Atkin et al. 2006). Likewise, elevated levels of UPR markers are seen in CSF and spinal cords of sALS patients (Atkin et al. 2008). Motor neuron-like cell lines, as well as primary spinal motor neurons, show increased levels of ER stress markers when subjected to CSF from ALS patients (Vijayalakshmi et al. 2011). UPR is initially a protective response, correcting mis-folded proteins, but the prolonged activation as seen in ALS, leads to the activation of apoptotic pathways (Hitomi et al. 2004).

Endosomal vesicle transport defects have been associated with ALS by the identification of fALS mutations in genes coding for proteins with function in the endosomal transport machinery. Mutations in Alsin, a guanine nucleotide exchange factor for small GTPases, are responsible for a form of juvenile onset ALS (ALS2 locus). Alsin takes part in endosomal fusion and trafficking as well as neurite outgrowth (Lai et al. 2006). In cultured neurons the loss of Alsin leads to increased susceptibility to glutamate-induced excitotoxicity, most likely due to an altered composition of AMPA-receptors (Lai et al. 2006) and thus suggesting a connection between vesicle transport defects and excitotoxicity. Several relatively rare mutations are also connecting trafficking defects and ALS. The vesicle-associated membrane binding protein-associated protein B (VAPB; ALS8 locus) is involved in vesicle transport and mainly located in the ER. VAPB aggregation is speculated to lead to ER stress and disruption of proteasome function and thus might contribute to an altered protein homeostasis and ultimately to motor neuron death (Suzuki et al. 2009). In addition, VAPB is involved in the UPR response (Nishimura et al. 2004; Moumen et al. 2011; Chen et al. 2010). Mutations in optineurin (ALS12 locus), a protein involved in the maintenance of the Golgi complex, exocytosis and various aspects of Golgi trafficking, lead to increased NF-κB activation, which is also upregulated in sALS and thus, might be connected with motor neuronal cell death (Maruyama et al. 2010). Charged multivesicular body protein 2B (CHMP2B), a component of the endosomal sorting complex, causes vacuolisation, lysosomal mis-localisation and impaired autophagy in cultured cells and has been associated with frontotemporal dementia (FTD; FTD3) and ALS (ALS17) (Parkinson et al. 2006; Cox et al. 2010). FIG4 gene (ALS11 locus) encodes a polyphosphoinositide phosphatase, which regulates the level of phosphatidylinositol 3,5 biphosphate, a signal lipid regulating the retrograde vesicle transport from lysosomes and late endosomes to the Golgi apparatus (Chow et al. 2009). FIG4 mutations found in human fALS cases have shown to induce vacuolization in yeast (Chow et al. 2009).

Impaired axonal transport is a key feature of ALS (Ferraiuolo et al. 2011). Both anterograde and retrograde axonal transport are impaired in mutant SOD1 transgenic mice early in the disease progression. The mechanism behind axonal transport defects is unknown, but is likely to be cargo-specific (Williamson and Cleveland 1999; De Vos et al. 2007; Bilsland et al. 2010). Anterograde transport along microtubules depends on kinesin motor proteins, while retrograde transport depends on cytoplasmic dynein. It is possible that impaired mitochondrial function could be caused to some extent by a decreased mitochondrial transport along axons, which would lead to an increased mitochondrial aging, because the charging of mitochondria with nuclear-encoded mitochondrial proteins in the perikaryon is decreased. This in turn could reduce the energy available for general axonal transport and thereby might aggravate the transport defect and also affect other cargos (De Vos et al. 2007; Miller and Sheetz 2004). However, by increasing the axonal mobility of mitochondria in mutant SOD1 transgenic mice, no beneficial effect on the neurodegeneration could be observed (Zhu and Sheng 2011). In mutant SOD1 transgenic mice, levels of tumor necrosis factor (TNF) are elevated, which can disrupt kinesin function through a mechanism involving p38-MAPK (Kiaei et al. 2007; De Vos et al. 2007) and glutamate has been shown to reduce axonal transport of neurofilament medium chain (NF-M) by activating protein kinases, which phosphorylate NFM proteins (Ackerley et al. 2000). Neurofilament hyperphosphorylation leads to decreased neurofilament transport and might also aggravate neurofilament aggregation in the perikaryon.

Transcription and RNA processing is altered in ALS (Ferraiuolo et al. 2011). TDP-43 (ALS10 locus) is involved in transcriptional regulation, alternative splicing and miRNA processing and FUS (ALS6 locus) is involved in transcriptional regulation, RNA and miRNA processing, and mRNA transport (Mackenzie et al. 2010). Under normal circumstances both are located in the nucleus (Mackenzie et al. 2010). Mutations in TDP-43 and FUS both account for 4 % of fALS cases. In sALS TDP-43 mutations are found in 1.5 % of the cases, while FUS mutations are found in <1 % (Mackenzie et al. 2010). ALS-associated mutations in TDP-43 and FUS lead to loss of nuclear localization, re-localization to the cytoplasm, and inclusion in cytoplasmic stress granules—a rapid, reversible cellular response to stress, controlling RNA metabolism (Liu-Yesucevitz et al. 2010; Ito et al. 2011; Dormann et al. 2010). How altered mRNA transcription and processing leads to motor neuron injury is unknown. It is possible that TDP-43 and FUS could be part of an RNA transport complex and that mutations in either could lead to the loss of axonal transport (Ferraiuolo et al. 2011). Another possibility is that the loss of nuclear expression leads to partial disruption of the RNA machinery, such as pre-mRNA splicing, nuclear mRNA export, mRNA sorting and processing of non-coding RNA (Ferraiuolo et al. 2011). A third option is that mutated TDP-43 and FUS-induced stress granules revert more slowly, leading to abnormal accumulation (Liu-Yesucevitz et al. 2010; Ito et al. 2011; Dormann et al. 2010). Angiogenin (ANG; ALS9) and Senataxin (SETX, ALS4) both have been associated with fALS (Greenway et al. 2006; Chen et al. 2004) and are also involved in RNA metabolism.

Neuroinflammation is a hallmark of ALS and involves glia activation and infiltration of peripheral immune cells, recently reviewed by (Papadimitriou et al. 2010). Similar to the ER stress response, the neuroinflammatory processes appear to have both protective and harmful effects on the neurodegeneration (Liao et al. 2012). Proinflammatory cytokines have been reported in the CSF of ALS patients (Kuhle et al. 2009) and evidence from mutant SOD1 transgenic mice lacking CD4, which develop an aggravated neurodegeneration (Beers et al. 2008), indicate that the inflammatory reactions have an impact on the ALS neurodegeneration. In chimeric mutant SOD1 transgenic mice, normal motor neurons display signs of ALS pathology when surrounded by mutant SOD1 expressing glial cells (Clement et al. 2003). Astrocytes expressing mutant SOD1 exhibit toxic effects on cultured primary motor neurons and motor neurons derived from both human and murine stem cells (Di Giorgio et al. 2008; Nagai et al. 2007). However, it is still under debate to what extent astrogliosis and microgliosis have beneficial and/or harmful effects on ALS pathology (Papadimitriou et al. 2010).

ALS appears to be a complex disorder and many factors contribute to the pathology. So far, mutations in 18 ALS genes/loci have been found to cause familial ALS (Table 1) and further genetic risk factors might contribute to sporadic ALS. Most of the ALS genes are ubiquitously expressed and involved in fundamental cellular processes, which raises the question why motor neurons are more vulnerable than other cells. The intuitive explanation that motor neurons are specifically vulnerable due to their extraordinary axon lengths is probably much too simple, since motor neurons degenerating first are not necessarily those with the longest axons and sensory neurons with similar axon lengths are not affected. However, motor neurons have shown to be particularly sensitive to glutamate excitotoxicity (Williams et al. 1997; Ince et al. 1993) as well as ER stress (Saxena et al. 2009). Motor neurons also seem to have a high threshold for mounting a heat shock response, a high sensitivity to oxidative damage and calcium overload via mitochondria (Sullivan et al. 2004; Panov et al. 2011). However, it is still poorly understood what makes motor neurons more susceptible than other cells.

The comprehension of the molecular processes underlying ALS is crucial for the development of an efficient treatment of the disease. Thus, the generation and investigation of suitable animal models (Table 2) contributes to both understanding the pathomechanisms and the developing therapeutic intervention.

## The wobbler mouse

Several mouse models for studying ALS exist (Table 2). The most commonly used is the SOD1^G93A^, which over-expresses a mutated human SOD1-gene (Gurney et al. 1994), but several animal models exist, where the SOD1 gene is mutated at different positions (Tovar et al. 2009). Recently, transgenic mouse models of Alsin- (knock out) and hTDP-43-associated fALS (transgenic) have been created (Tovar et al. 2009; Shan et al. 2010; Wils et al. 2010; Wegorzewska et al. 2009). However, the majority of ALS cases are sporadic with unknown cause; even though clinically undistinguishable from fALS this fact indicates the need for further animal models.

The wobbler mouse arose spontaneously in a C57BL/Fa strain and was first described by Falconer in the late 1950s (Falconer 1956). The autosomal recessive-mutation was soon linked to motor neuron degeneration and is caused by a point mutation in the *Vps54* gene (Schmitt-John et al. 2005). The wobbler mouse is the best-characterized spontaneous mutant with motor neuron degeneration, which mimics several of the features seen in ALS patients, but where the comparable mutation of VPS54 has not been found in ALS patients so far, although only a limited number of patients have been examined for the mutation (Meisler et al. 2008).

## The wobbler phenotype

When describing the disease symptoms of the wobbler mouse it is convenient to divide them into three different phases, based on the development of physical symptoms: the pre-symptomatic, the evolutionary and the stabilized phase (Boillee et al. 2003). The pre-symptomatic phase lasts from birth to around 3 weeks of age. At this point homozygous wobbler mice exhibit little to no clinical symptoms; in homozygous wobbler mice body weight, grip strength, righting reflexes and grid-walking tests all appear normal (Boillee et al. 2003). During the evolutionary phase, which lasts from around 3 weeks to 3 months of age, clinical, morphological and molecular symptoms develop. Homozygous wobbler mice display reduced bodyweight and muscle strength; develop a wobbly gait and head tremor. Muscle atrophy leads to a pointed muzzle, and suspension by the tail results in flexed instead of extended hind limbs (Boillee et al. 2003). The stabilized phase, from 3 months of age to death, is characterized by an arrest in the progression of motor neuron degeneration (Boillee et al. 2003). In addition, male homozygous wobbler mice are infertile due to failed spermatogenesis (Leestma and Sepsenwol 1980; Heimann et al. 1991), which will be discussed later.

First morphological changes appear already at the pre-symptomatic stage. Affected motor neurons are characterized by weak staining of Nissl bodies and enlarged somas (Duchen and Strich 1968; Blondet et al. 2002), although the number of motor neurons remains unaffected in the median nerve nuclei of the cervical spinal cord at this stage (Blondet et al. 2002). In the brainstem, the ventral reticular magnocellular nucleus, and the motor nuclei of the central nerves V and VII, cells with diverse anomalies are found but rarely elsewhere in the spinal cord or brain (Duchen and Strich 1968). Schwann cells display intra-axonal invaginations, dilation of the ER is seen and motor neurons start to display vacuolization (Mitsumoto and Bradley 1982). Affected motor neurons are characterized by microtubule segregation and the presence of large, dense secondary lysozymes (Mitsumoto and Bradley 1982). First signs of neurodegeneration are observed on day 13 post natal (p.n.) in the thalamus (*N. ventralis*), deep cerebellar nuclei, brain stem (*N. vestibularis*) and spinal inter neurons, while spinal motor neurons follow around day 15 p.n. (Rathke-Hartlieb et al. 1999). Neuroinflammation is seen as consequence of the neurodegeneration; from day 17 p.n. onwards, reactive astrocytes are seen and around day 23 p.n. microgliosis is observed (Rathke-Hartlieb et al. 1999). In the motor cortex of wobbler mice a reduced number of parvalbumin-positive GABAergic interneurons is seen at the pre-symptomatic phase between day 15 and 25 (Nieto-Gonzalez et al. 2011). Thus, neurodegeneration is not restricted to motor neurons in the wobbler mouse. Interestingly, a reduced release of GABA is seen in synaptosomes isolated from the cervical spinal cord of 12-week-old wobbler mice (Bonanno et al. 2009), indicating that GABAergic interneurons might play a critical role in the wobbler motor neurodegeneration.

During the evolutionary phase numerous signs of degeneration and a marked loss of motor neurons become apparent (Pollin et al. 1990). In the brain degenerating motor neurons show weak staining of Nissl bodies without any vacuoles being present, as well as eccentric localization of nuclei (LaVail et al. 1987). At this point, the 3-month-old wobbler mouse displays signs of muscular denervation in the forelimbs, though not in the hind limbs (Andrews et al. 1974). There is a reduction of motor nerve terminals and axonal sprouts (Duchen and Strich 1968; LaVail et al. 1987) together with reduced diameter or even loss of myelin sheaths, resulting in large non-myelinated fibres (Bird et al. 1971; Biscoe and Lewkowicz 1982; Lewkowicz 1979). At the end of the evolutionary phase, reactive astrocytes and microglial cells are present throughout the dorsal and ventral horn of the spinal cord (Boillee et al. 2001). Muscles in wobbler mice show a shift from a slow oxidative to fast glycolytic myosin heavy chain isoform pattern expression (Agbulut et al. 2004; Staunton et al. 2011).

On the molecular level, abnormal aggregation of proteins such as neurofilaments occurs in the cytoplasm (Pernas-Alonso et al. 2001). As a result of the decrease in chaperone protein number, reactive-ubiquitin deposits are seen (Boillee et al. 2003). During the evolutionary phase, large and numerous vacuoles appear in the cell body of affected motor neurons until they fill up the soma. The vacuoles are thought to originate from dilated ER, but are of otherwise unknown composition (Mitsumoto and Bradley 1982). Recent ultrastructural analyses suggest that the vacuoles first appear in the proximity of the Golgi apparatus and are in later stages ER-derived (Palmisano et al. 2011). Enlarged APP- and Rab7-positive endosomal structures become apparent, which might be identical with the early vacuoles seen on electron micrographs (Palmisano et al. 2011). Similar effects were found in a subset of sALS patients and might indicate endosomal vesicle transport defects (Palmisano et al. 2011).

Proteomic profiling has been conducted on both spinal cord and muscles from wobbler mice. At 4 weeks of age, the cervical part of the spinal cord is affected while the lumbar part remains unaffected. In the cervical- and lumbar spinal cord of individuals, homozygous for the wild-type allele of *Vps54*, a divergent protein pattern was found, while in wobbler individuals the protein pattern of cervical and lumbar spinal cord was similar (Bastone et al. 2009). The wobbler cervical spinal cord shows alterations of proteins involved in the glutamate–glutamine cycle, energy transduction, astrogliosis or redox functions, when compared to wild-type cervical spinal cords. In the lumbar spinal cord of wobbler mice, proteins involved in vesicle trafficking are upregulated when compared to wild-type lumbar spinal cords (Bastone et al. 2009).

Impaired axonal transport, a key feature of ALS, is observed in wobbler mice as well. Slow- and fast anterograde- as well as fast retrograde axonal transport of proteins is affected, both with respect to speed and the quantity of proteins transported (Bird et al. 1971; Mitsumoto and Gambetti 1986; Mitsumoto et al. 1990, 1993).

As mentioned earlier, alterations in mitochondria and mitochondrial dysfunction play a critical role in ALS. In wobbler mice mitochondrial abnormalities have been reported from onset of disease symptoms, comprising altered oxygen consumption rates and morphological abnormalities (Xu et al. 2001; Santoro et al. 2004; Dave et al. 2003a). It is possible that an increased activation of protein kinase C delta (PKC-delta) could induce mitochondrial apoptosis in wobbler motor neurons (Dave et al. 2005).

TDP-43 expression is increased in the spinal cord of wobbler mice. Like in ALS, displacement of TDP-43 from the nucleus to the cytoplasm is seen, as well as TDP-43 inclusion in ubiquitin-positive aggregates (Dennis and Citron 2009). In general, motor neurons display an abnormal RNA metabolism with a reduced RNA content (Murakami et al. 1981), indicating alterations in transcription and RNA processing. In accordance with this, an altered protein expression profile is seen not only in affected motor neurons, but also in completely healthy motor neurons (Murakami et al. 1980).

The involvement of glutamate excitotoxicity in the wobbler disease presents a murky picture. Glutamate levels in the brain of wobbler mice were found to be similar to levels in controls and even slightly downregulated in the spinal cord (Krieger et al. 1991). On the other hand, neural precursor cell (NPC)-derived astrocytes were found to release excessive levels of glutamate into the medium in cell culture while at the same time having lower concentrations of intracellular glutamate (Diana et al. 2010). Binding to the metabotropic glutamate receptor was found to be unaltered whereas the binding to NMDA receptors, Kainate-receptors and AMPA-receptors was increased in the wobbler cervical spinal cord (Tomiyama et al. 1994). In the cervical spinal cord from wobbler mice a reduced level of neuronal glutamate receptor EAAC1 has been shown, while the glial glutamate transporters GLT-1 and GLAST were shown in normal levels (Bigini et al. 2001). In vivo, grafted astrocytes derived from NPCs showed reduced immunoreactivity for both GLT-1 and GLAST (Diana et al. 2010). These astrocytes also displayed a reduced uptake of d-[2,3-^3^H]-aspartic acid most likely due to a lower number of glutamate receptors (Diana et al. 2010). In synaptosomes isolated from the cervical spinal cords of 4- and 12-week-old wobbler mice, the glutamate uptake was shown not to be altered while the release of [^3^H]-d-aspartate was increased (Bonanno et al. 2009). However, the role of glutamate excitotoxicity in the wobbler MND remains largely unclear.

The neurological phenotype of the wobbler mouse resembles ALS in many ways as summarized in Table 3; and thus, the wobbler mouse is a valuable animal model to investigate the molecular and cellular mechanisms underlying the pathologic changes.

**Table 3:** Phenotypic aspects of wobbler mice compared to sALS and fALS

	Effects, cellular effects/affected processes		Wobbler/ref		ALS/ref
Effects on the organism	Motor defects	**+**	(Duchen and Strich 1968)	**+**	(Bruijn et al. 2004)^a^
	Tremor, hyperreflexia, spasticity	**+**	(Duchen and Strich 1968)	**+**	(Bruijn et al. 2004)^a^
	Muscle weakness, cramps	**+**	(Duchen and Strich 1968)	**+**	(Bruijn et al. 2004)^a^
	Cognitive defects, frontotemporal dementia	**–**	Not tested	**±**	In some cases (Achi and Rudnicki 2012)^a^
	Death due to respiratory failure	**+**	(Duchen and Strich 1968)	**+**	(Bruijn et al. 2004)^a^
Effects on cells	Degeneration of upper and lower motor neurons	**+**	(Duchen and Strich 1968)	**+**	(Ferraiuolo et al. 2011)^a^
	Astrogliosis	**+**	(Duchen and Strich 1968)	**+**	(Ferraiuolo et al. 2011)^a^
	Microgliosis	**+**	(Duchen and Strich 1968)	**+**	(Ferraiuolo et al. 2011)^a^
	Muscle atrophy	**+**	(Duchen and Strich 1968)	**+**	(Ferraiuolo et al. 2011)^a^
	Hyperexcitability, decreased GABAergic inhibition	**+**	(Nieto-Gonzalez et al. 2011)	**+**	(Vucic et al. 2008)
	Spermatogenesis defect	**+**	(Heimann et al. 1991)	**–**	Not reported
Effects in motor neurons	Vesicle traffic defects	**+**	(Palmisano et al. 2011)	**+**	(Ferraiuolo et al. 2011)^a^
	Enlarged endosomes vacuolization	**+**	(Palmisano et al. 2011)	**+**	(Palmisano et al. 2011)
	Impaired axonal transport	**+**	(Mitsumoto et al. 1990)	**+**	(Williamson and Cleveland 1999)
	Protein missorting	**+**	(Perez-Victoria et al. 2008)	**+**	(Yang et al. 2001; Nishimura et al. 2004)
	Ubiquitin-positive protein aggregates	**+**	(Dennis and Citron 2009)	**+**	(Neumann et al. 2006)
	TDP-43-positive protein aggregates	**+**	(Dennis and Citron 2009)	**+**	(Neumann et al. 2006)
	Neurofilament aggregations (perinuclear)	**+**	(Pernas-Alonso et al. 2001)	**+**	(Hirano et al. 1984)
	Mitochondrial dysfunction	**+**	(Santoro et al. 2004)	**+**	(Wiedemann et al. 2002)
	Cortical hyperexcitability/excitotoxicity	**+**	(Nieto-Gonzalez et al. 2011)	**+**	(Vucic et al. 2008)

^a^Effects, which were several times reported, but recently reviewed in

## Towards understanding the disease mechanism

In order to understand the disease mechanism, it has been crucial to identify the gene affected by the *wobbler* mutation. The recessive inheritance indicates a loss-of-function effect and the investigation of chimeric mice pointed to a cell-autonomous effect of the *wobbler* mutation (Augustin et al. 1997).

## Identification of the wobbler gene, *Vps54*

The *wobbler* mutation was mapped to the proximal mouse chromosome 11 by backcrossing to various mouse strains (Kaupmann et al. 1992; Resch et al. 1998; Fuchs et al. 2002). The homologous region in the human genome was found to be on chromosome 2p13 (Korthaus et al. 1997) and physical maps of the proximal mouse Chr 11 and the corresponding region on 2p13 were established (Resch et al. 1998; Fuchs et al. 2002). The critical interval was narrowed down to less than 1 cM, corresponding to 894 kb (Schmitt-John et al. 2005). All genes in the critical interval were sequenced and finally a single base pair exchange was found in the last exon of the *Vps54* gene (Schmitt-John et al. 2005).

**Fig. 1:**
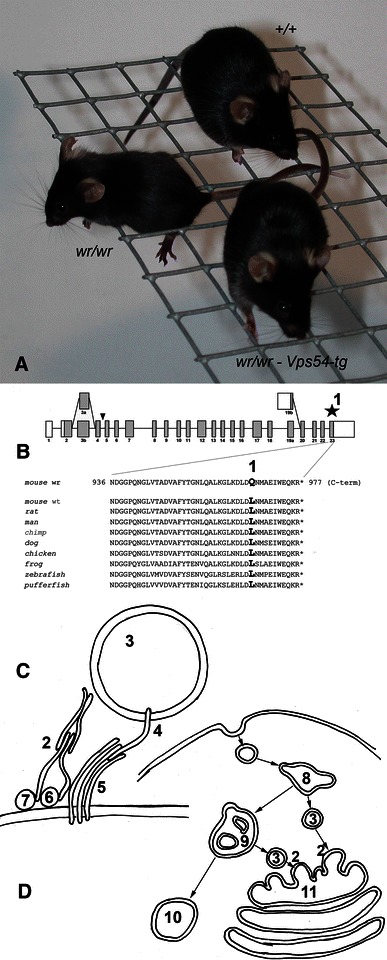
The wobbler phenotype is caused by to the partial loss of GARP function. **a** Wild type- (+/+), wobbler mouse (*wr*/*wr*) with motor deficits, and a transgenic rescued mouse (wr/wr–Vps54) with a wild-type Vps54 transgene compensating the motor defect (Schmitt-John et al. 2005). **b** Schematic drawing of the *Vps54* gene and amino acid sequence of the C-terminus of Vps54 proteins from various species. (*1)* indicates the wobbler point mutation in exon 23 of Vps54 leading to a glutamine instead of a conserved leucine. **c** Function of the GARP complex (*2)* in tethering early and late endosome-derived vesicles (*3)* to the TGN *11*. The GARP complex consisting of Vps51, Vps52, Vps53 and Vps54 interacts with Rab6 (*6*) and Arl1 (*7)* and mediates vSNARE (*4)–*tSNARE (*5)* mediated fusion of the vesicle and target membrane. **d** The GARP complex (*2)* functions in the retrograde vesicle transport. Endocytic vesicles reach early endosomes (*8)* then late endosomes (*9)* and further to lysosomes (*10)*. Alternatively, early and late endosome-derived vesicles (*3)* can be retrogradely transported to the TGN (*11)*, where the GARP complex (*2)* is localized. The wobbler mutation destabilizes Vps54 and thereby the whole GARP complex and thus leads to a partial loss of GARP function and impairments of the retrograde vesicle traffic

Vps54 (vacuolar protein sorting 54) is a component of the Golgi-associated retrograde protein (GARP) complex (Fig. 1). The *Vps54* gene is located on proximal chromosome (11 21,139,284-21,221,236 [Ensembl; ENSMUSG00000020128]). Four splice isoforms of the gene were detected, where the predominant encodes a protein of 977 amino acids (Schmitt-John et al. 2005). The *wobbler* mutation was identified in exon 23; here, an A → T transversion at the second position of codon 967 was found (Schmitt-John et al. 2005). This transversion results in an amino acid substitution, where the conserved leucine at position 967 is exchanged by a glutamate (Schmitt-John et al. 2005).

To ascertain that the *Vps54* gene indeed is affected by the *wobbler* mutation, a transgenic rescue experiment was conducted. A transgene covering the full genomic sequence of wild-type *Vps54* including sequences 63 kb upstream and 18 kb downstream was used to generate transgenic mice, which were bred into the wobbler strain. This wild-type *Vps54* transgene could compensate the wobbler phenotype (Schmitt-John et al. 2005), wild-type *Vps54* transgenic *wr/wr* individuals had normal grip strength, mobility and bodyweight. Likewise, histological appearance of motor neurons and astrogliosis were comparable to wild-type control mice. Male rescued mice were also able to reproduce normally and father viable offspring (Schmitt-John et al. 2005). This proves that *Vps54* is the *wobbler* gene and demonstrates that the *wobbler* point mutation of Vps54 is responsible for both the neurological and the spermatogenesis phenotype.

The *wobbler* point mutation of Vps54 was expected to be a hypomorphic allele of *Vps54*, but not a complete loss-of-function. Thus, a null-mutant allele was generated using a gene trap embryonic stem cell line (Schmitt-John et al. 2005). The *Vps54* gene trap clone, *Vps54* ^*gt(pGT10)2841Ucd*^, hence termed as *Vps54* ^*β-geo*^, has a β-geo (β-galactosidase-neomycin resistance fusion protein) cassette inserted between exon 4 and 5, and the non-functional fusion protein product contains the first 152 amino acids of Vps54 fused with β-geo. The embryonic stem cells were used to generate chimeric mice and among their offspring heterozygous *Vps54* ^*β-geo/*+^ mice were obtained, which generally had a normal phenotype (Schmitt-John et al. 2005). Matings of heterozygous *Vps54* ^*β-geo/*+^ mice failed to produce any homozygous *Vps54* ^*β*-*geo/β*-*geo*^ pups. *Vps54* ^*β*-*geo/β*-*geo*^ embryos were found severely developmentally retarded at E11.5, and at E12.5 only resorption sites with *Vps54* ^*β*-*geo/β*-*geo*^ genotype were found, thus, Vps54-null mutation causes embryonic lethality around day E11.5 (Schmitt-John et al. 2005). When examining retarded *Vps54* ^*β*-*geo/β*-*geo*^ embryos at E11.5 it is seen that the spinal cord is underdeveloped, the dorsal root ganglia are nearly absent and severe hypoplasia is seen in the atrial and ventricular myocardium (Schmitt-John et al. 2005). *Vps54* ^*β*-*geo/wr*^ compound heterozygotes display a standard wobbler phenotype with MND and spermatogenesis defect, indicating that one point mutant *Vps54* allele is sufficient to rescue embryonic lethality (Schmitt-John et al. 2005).

## The GARP complex in vesicle tethering

The positional cloning of the *wobbler* gene has connected the ALS-like wobbler motor neurodegeneration to the partial loss-of-Vps54 function (Schmitt-John et al. 2005) and thereby to the GARP complex, of which Vps54 is a component (Fig. 1). The function of the GARP complex has recently been reviewed by Bonifacino and Hierro (2011).

In yeast the GARP complex was described as a vesicle-tethering factor (Conibear and Stevens 2000; Whyte and Munro 2002),. The GARP complex is a member of the family of multisubunit tethering complexes (MTC) and consists of the four subunits Vps51p, Vps52p, Vps53p and Vps54p in a 1:1:1:1 stoichiometric ratio (Conibear et al. 2003). Due to sequence similarities and structural relation the GARP complex belongs to the CATCHR (complexes associated with tethering containing helical rods) group of MTCs also comprising Dsl1-, COG- and Exocyst complexes [reviewed in Bonifacino and Hierro (2011)]. The complex is involved in intracellular vesicular trafficking and tethers vesicles derived from both early and late endosomes to the trans Golgi network (TGN) (Quenneville et al. 2006; Conibear et al. 2003). In yeast the C- and N-terminal domains of Vps54p were shown to have different functions. The N-terminal domain is important for GARP complex assembly and stabilization, while the C-terminal domain facilitates localization to an early endocytic compartment (Quenneville et al. 2006). Point mutations of highly conserved nucleotides in the C-terminal region of Vps54 do not affect retrograde transport from late endosomes, but block early endosome recycling by preventing localization to the polarized early endosome (Quenneville et al. 2006).

There is a high degree of conservation between yeast and mammalian GARP complexes. However, in yeast, knock out of any of the subunits is non-lethal (Conibear et al. 2003), while in mice at least-null mutation of either Vps54 (Schmitt-John et al. 2005) or Vps53 (Schmitt-John and Moser, unpublished results) causes embryonic lethality around day 11 of the embryonic development and thus might be considered a GARP-null mutation. Even though Vps54- and Vps53-null mutant embryos die around day 11 of the embryonic development, blastocyst-derived embryonic stem cells and embryonic fibroblasts from 9.5-day-old embryos are able to grow in cell culture and can be maintained over several passages in cell culture (Schmitt-John and Moser, unpublished results).

Interestingly, Vps52-null mutation was recently associated with a recessive t-complex mutation (t^w5^-lethal), which causes a gastrulation defect and thus earlier embryonic lethality (Sugimoto et al. 2012). This might argue for an additional function of murine Vps52 in gastrulation, perhaps independent of the GARP complex.

The human GARP complex consists of VPS52, VPS53, VPS54 (Liewen et al. 2005) and ANG2, the latter corresponding to Vps51 in yeast (Perez-Victoria et al. 2010) making the tetrameric, 1:1:1:1 complex evolutionary conserved. RAB6, which targets vesicles between organelles, is an interaction partner of human VPS52 just like its yeast homolog Ypt6 interacts with Vps52 (Liewen et al. 2005). Syntaxin 10 also interacts with the GARP complex like Tlg1P in yeast (Liewen et al. 2005). Other SNAREs, syntaxin 6, syntaxin 16 and Vamp4 have been shown to interact with the mammalian GARP complex in a direct manner (Perez-Victoria and Bonifacino 2009). The GARP complex seems not only to bind to these SNAREs, but also to promote their assembly into complexes and regulating their correct localization (Perez-Victoria and Bonifacino 2009). It was also shown that the tethering function of the GARP complex is not dependent on the interaction of the GARP complex with SNAREs (Perez-Victoria and Bonifacino 2009). It has been shown that the human GARP complex is required for the mannose 6-phosphate-receptor-dependent sorting of cathepsin D (CatD) to lysosomes (Perez-Victoria et al. 2008). At the TGN mannose-6-phosphate modified hydrolases bind to cation-dependent- (CD-MPR) and cation-independent mannose-6-phosphate receptors (CI-MPR) (Ghosh et al. 2003) and the complexes are delivered to endosomes by transport carriers, where the hydrolases are released and continue to lysosomes, while the MPRs returned to the TGN (Ghosh et al. 2003). By RNA interference-based depletion of subunits of the GARP complex, it was shown that due to the impaired MPR recycling, CatD was released into the medium of cultured cells and leading to swollen lysosomes (Perez-Victoria et al. 2008). Vps54 with the *wobbler* point mutation was shown to be able to assemble into the GARP complex and restore the mis-distribution of CatD and CI-MPR (Perez-Victoria et al. 2008). The recently determined structure of the C-terminal part of the murine Vps54-protein covering 145 amino acids (from residue 836 to 977) including the residue where the *wobbler* mutation is located shows similarities to specific domains seen in other tethering complexes, such as the exocyst-, Dsl1- and COG-complexes (Perez-Victoria et al. 2010). The critical residue, 967, seems to be involved in several hydrophobic interactions with other residues. The *wobbler* mutation from leucine to glutamate does not change the volume of the residue, but it alters the hydrophobicity of the side chain and thus is thought to cause a destabilization of Vps54 and probably the whole GARP complex (Perez-Victoria et al. 2010). The wobbler amino acid exchange has been shown to cause a decreased level of Vps54 protein in several tissues of wobbler mice along with decreased levels of Vps53 protein (Perez-Victoria et al. 2010), indicating a higher rate of degradation of not only Vps54, but the whole GARP complex and thus, limiting the availability of the GARP complex for tethering endosome-derived vesicles to the TGN (Perez-Victoria et al. 2010). Since wobbler mice are viable, it is not surprising that the wobbler version of Vps54 is able to maintain some functionality or that even a low level of the GARP complex is able to uphold retrograde vesicle transport to some degree. However, in wobbler cells impairments of the retrograde vesicle transport and missorting of proteins are seen (Schmitt-John, Moser, unpublished data).

## Cellular consequences of the wobbler mutation

**Fig. 2:**
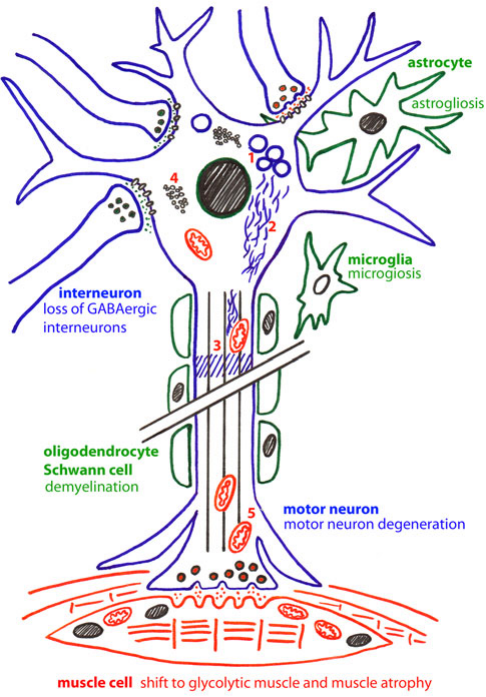
Cellular effects of the *wobbler* mutation. The schematic drawing show a lower motor neuron connected to skeletal muscle cells and associated with interneurons and glial cells, such as astrocytes, microglial cells, oligodendrocytes and Schwann cells. The effects on the different cells, motor neuron degeneration muscle atrophy, astrogliosis, microgliosis and the loss of GABAergic interneurons are indicated. The effects of the dysfunction of cellular processes are given with numbers: (*1)* the formation of APP- and Rab7-positive vacuoles, (*2)* neurofilament aggregations, (*3)* impaired axonal transport, (*4)* further ubiquitin-positive protein aggregates and mitochondrial dysfunction (*5)*

The cellular effects of the *wobbler* mutation are summarized in Fig. 2. As reported above, the ALS-like wobbler phenotype is caused by a point mutation of Vps54, which leads to destabilization of Vps54 protein and the whole GARP complex and thereby to impairments of the retrograde vesicle transport. Thus, we have a relatively clear conception of the primary cause of the disease and the final effects like muscle atrophy, astrogliosis and microgliosis can be seen as a logical consequence of the progressive motor neuron degeneration. However, the link between GARP malfunction and motor neuron degeneration is still missing.

One of the obvious questions is: Why does the mutation of a ubiquitously expressed gene, *Vps54*, affect predominantly motor neurons and perhaps a few other types of neurons? Ubiquitous- or at least widespread expression is a feature shared with most of the human fALS disease genes and it was frequently speculated that the extraordinary length of motor neurons might make them more susceptible to minor impairments of cellular processes. However, sensory neurons can have similar lengths and are not affected and in wobbler mice cranial and cervical motor neurons are the first affected ones, but they are not necessarily the longest. Thus, we have to expect a more complex explanation for this hitherto open question.

Starting from the solid ground of Vps54 being affected by the *wobbler* mutation (Schmitt-John et al. 2005), leading to a decreased stability and abundance of the GARP complex (Perez-Victoria et al. 2010) in all cells of wobbler mice, we can assume an impairment of the retrograde vesicle transport from endosomal compartments to the TGN. This surely affects the sorting of proteins like MPRs and lysosomal proteins (Perez-Victoria and Bonifacino 2009) and probably several other proteins. This vesicle transport defect might also explain the accumulation of enlarged APP-positive endosomal structures, or vacuoles seen in degenerating wobbler motor neurons and in a subset of human sALS cases (Palmisano et al. 2011). The impaired retrograde vesicle transport might be responsible for the increasing size of the endosomal compartments, probably leading to a critical imbalance in the membrane distribution and thereby probably affecting all kinds of intracellular transport. Defects in the fast anterograde (Mitsumoto et al. 1993) and retrograde axonal transport (Mitsumoto et al. 1990) have been reported for wobbler neurons, perhaps as a consequence of general transport problems. Impairments of the cellular transport systems could also explain perinuclear neurofilament aggregation seen in wobbler mice (Pernas-Alonso et al. 1996; Schmitt-John, Moser, unpublished data) but also in ALS patients (Hirano et al. 1984).

Impairments of the cellular vesicle transport processes are also expected for fALS (ALS2, ALS8, ALS11, and ALS17) and thus might be a more general hallmark of ALS. Transport defects might also explain the mitochondrial dysfunction seen in wobbler cells (Santoro et al. 2004). Impaired transport of mitochondria to the cell body and subsequently decreased re-loading with newly synthesized nucleus-encoded mitochondrial proteins might lead to dysfunction and premature aging of mitochondria. This might be especially critical in the distant periphery at the neuro-muscular junction. Mitochondrial dysfunction, transport deficits and perhaps lysosomal degradation defects (due to missorted lysosomal proteins) all might contribute to the accumulation of ubiquitinylated protein aggregates including TDP-43 found wobbler motor neurons (Dennis and Citron 2009).

Protein missorting might be most critical for motor neuron survival when survival factor receptors like tyrosine receptor kinases (TRK) or ion channels on the neuronal surface are affected, which would directly affect the electrical properties of the neurons. Recent electrophysiological recordings have demonstrated a hyperexcitability of layer V pyramidal neurons of the wobbler motor cortex already prior to the onset of disease symptoms, due to decreased GABAergic inhibition (Nieto-Gonzalez et al. 2011). Similar cortical hyperexcitability has also been reported as an early feature of human ALS (Vucic et al. 2008, 2009) and recently decreased GABA levels have been found in the motor cortex of ALS patients (Foerster et al. 2012). Intuitively one might expect that the cortical hyperexcitability in wobbler mice is caused by missorting of GABA receptors in wobbler principal neurons, but in contrast a decreased number of GABAergic interneurons in the wobbler motor cortex was found (Nieto-Gonzalez et al. 2011). Thus, the decreasing numbers of GABAergic interneurons are a very early event in the progression of the wobbler MND, already evident in the pre-symptomatic phase; an effect not easily connected to the reported vesicle transport defects. However, the loss of short interval intracortical inhibition seen in ALS patients has been attributed to the degeneration of inhibitory GABAergic interneurons (Vucic et al. 2009), which suggests the wobbler mouse for the investigation of this aspect.

Taken together, the wobbler motor neuron degeneration shows most of the features reported for sALS and fALS (Table 3), such as, mitochondrial dysfunction, transport defects, protein aggregation but also cortical hyperexcitability making it a valuable ALS animal model.

Muscle atrophy (Duchen and Strich 1968) and the neuroinflammatory processes (Rathke-Hartlieb et al. 1999) like astrogliosis and microgliosis are well described for the wobbler mouse and closely resemble human ALS. These effects are thought to be secondary effects of the neurodegeneration. However, it is still under debate to what extent neuroinflammation is beneficial and/or harmful for motor neuron survival in ALS (Philips and Robberecht 2011) and the wobbler mouse. Muscle atrophy is doubtless a logical consequence of the loss of motor neurons. Recent proteome analysis of wobbler muscle suggests a transition from slow to fast muscles and thus resembles muscle atrophy caused by denervation or disuse (Staunton et al. 2011).

The cellular consequences of the *wobbler* mutation, like those of sALS and fALS, are still unclear. How exactly the vesicle transport defects, directly or indirectly cause the observed effects like protein missorting, protein aggregation, vacuolization, mitochondrial dysfunction and hyperexcitability and how these finally lead to motor neuron death and in which way other cells like astrocytes, microglial cells or interneurons contribute remains elusive.

## Wobbler spermatogenesis defect

When discussing the cellular effects of the *wobbler* mutation, spermatogenesis cannot be excluded. Unlike human MNDs, the *wobbler* mutation has a pleiotropic effect on the spermatogenesis. Homozygous wobbler males are sterile and produce decreased numbers (oligospermia) and abnormally shaped round-headed sperm cells (globozoospermia) with decreased motility (Heimann et al. 1991). The positional cloning of the *wobbler* gene and the subsequent transgenic rescue of the wobbler phenotype (Schmitt-John et al. 2005) clearly demonstrated that the wobbler point mutation of Vps54 causes both, the motor neurodegeneration and the spermatogenesis defect and thus suggests a role for Vps54 and probably the GARP complex in spermatogenesis.

Spermatogenesis is the formation of haploid sperm cells, spermatozoa from spermatogonia including meiosis and final differentiation of the haploid spermatids to the very specialized mature spermatozoa. The latter process, called spermiogenesis, is affected in wobbler males. Wobbler spermatozoa show defective acrosomes and incomplete condensation of the nucleus, similar to the defects seen in human globozoospermia (Heimann et al. 1991). In early spermiogenesis, the Golgi apparatus is extremely important. The proper formation of an acrosome depends on vesicles and granules produced from the Golgi apparatus. The Golgi-derived pro-acrosomal vesicles attach to the nuclear envelope and fuse to an acrosomic granule, which starts to flatten into a small cap over the nuclear surface. During the acrosome formation the spermatids start to become elongated and nuclei start to condense. In the case of wobbler males fusion of pro-acrosomal vesicles and nuclear condensation fails, leading to round-headed sperms with defective acrosome, reduced motility and midpiece defects (Heimann et al. 1991; Paiardi et al. 2011).

Vps54 has recently been shown to be involved in acrosome formation (Berruti et al. 2010); in spermatocytes the distribution of Vps54 is diffuse while during acrosome formation Vps54 concentrates at the developing acrosomal cap (Berruti et al. 2010). In this respect, Vps54 follows the route of the endosomal sorting complex, ESCRT-0 and USP8, a de-ubiquitinating enzyme. Male wobbler individuals fail to develop Vps54-positive vesicles during acrosome formation, Vps54 remains scattered in the cytoplasm and no USP8-positive acrosomal structures were found in wobbler testes (Paiardi et al. 2011). USP8 has been shown to be involved in endosomal sorting and is highly expressed not only in male germ cells, but also neuronal cells (Berruti and Martegani 2005; Bruzzone et al. 2008). This might suggest that endosomal traffic and acrosome formation share common features and that USP8, Vps54 and perhaps the GARP complex are important for both processes.

Globozoospermia is a rare male infertility disorder where in vitro fertilization fails due to the inability of the sperms to interact with the oocyte. Even intracytoplasmic sperm injection (ICSI) has little success, because the injected sperm cells fail to activate the oocyte. Since wobbler sperm resembles the human sperm defects and also fail to activate oocytes after ICSI, the wobbler mouse was used as an animal model to test methods for assisted oocyte activation (AOA). After injection of wobbler spermatozoa in mouse oocytes, AOA leads to successful fertilization (Heytens et al. 2010). Furthermore, it could be shown that the oocyte-activating factor PLCzeta is mis-localized on the surface of wobbler sperms, which could explain why wobbler sperms fail to activate oocytes after ICSI (Heytens et al. 2010). Thus, the wobbler mouse appears to be a useful animal model not only for ALS, but also globozoospermia and indicates shared features between acrosome formation and retrograde vesicle transport in neurons.

## Towards the treatment of ALS

The rapid and reproducible progression of symptoms and the possibility to easily monitor the evolution of motor dysfunction makes the wobbler mouse a reliable model to evaluate the efficacy of different pharmacological treatments.

In the last decades many different compounds have been tested in wobbler mice, summarized in Table 4. The beneficial effects achieved by treatments with a wide range of compounds further enforce the hypothesis of the heterogeneity of the “wobbler mouse disease”. This characteristic is an important point shared with human ALS (Beghi et al. 2007).

**Table 4:** List of therapeutic approaches tested on wobbler mice

Target	Compound(s)	Effect on WR MND	Reference
Trophic factors neuroprotection	BDNF (brain-derived neurotrophic factor)	Beneficial, delayed motor impairment, inhibition of NOS activity	(Mitsumoto et al. 1994; Tsuzaka et al. 2001)
	CTNF (ciliary neurotrophic factor)	Beneficial, delayed motor impairment	(Mitsumoto et al. 1994)
	BDNF + CTNF	Beneficial, delayed motor impairment	(Mitsumoto et al. 1994)
	Human recombinant IGF (insulin-like growth factor)	Beneficial, delayed motor impairment, increase of body weight	(Ikeda et al. 1995a)
	IGF + GAG (glycosaminoglycans)	Beneficial, delayed motor impairment, increase of body weight	(Gorio et al. 1998; Vergani et al. 1999)
	CT-1 (cardiotrophin-1)	Beneficial, delayed motor impairment	(Mitsumoto et al. 2001)
Antiinflammatory agents	LIF (leukaemia inhibitory factor)	Beneficial, increased muscle strength	(Ikeda et al. 1995a)
	IL-6 (interleukin-6)+ soluble IL6 receptor	Beneficial, delayed motor impairment	(Ikeda et al. 1996)
	Human tumor necrosis factor (TNF) binding protein 1	Beneficial, delayed motor impairment	(Bigini et al. 2008)
	VB3323, a TLR4 (Toll-like receptor 4) antagonist	Beneficial, delayed motor impairment, decreased microglia activation	(Fumagalli et al. 2006; De Paola et al. 2012)
	EPO (erythropoietin)	Beneficial, improved motor performance, decreased astro- and microgliosis	(Mennini et al. 2006)
Antiglutamatergic agents	Riluzole	Beneficial, delayed motor impairment	(Ishiyama et al. 2004)
	MK801, a NMDA receptor antagonist	No beneficial effects	(Krieger et al. 1992)
	RPR119990, an AMPA receptor antagonist	No beneficial effects	(Fumagalli et al. 2006)
Mitochondrial support and antioxidant agents	Creatine monohydrate	Beneficial, improved motor performance	(Ikeda et al. 2000a, b)
	HBOT (hyperbaric oxygen therapy)	Beneficial, improved motor performance	(Dave et al. 2003a, b)
	ALCAR (acetyl-l-carnitine	No beneficial effects	(Bigini et al. 2008; Beghi et al. 2011)
	OPC-14117 a free radical scavenger	Beneficial, improved motor performance, decreased formation of lipid peroxides	(Abe et al. 1997)
	Lecithinized superoxide dismutase	Beneficial, improved motor performance	(Ikeda et al. 1995b)
	l-NAME, a nonselective NOS inhibitor	Beneficial, improved motor performance	(Ikeda et al. 1998)
Steroid hormones	Progesterone	Beneficial, improved motor performance	(Gonzalez Deniselle et al. 2002; Deniselle et al. 2012)
	U-74389F, a 21-amino-steroid	Beneficial, improved motor performance and decreased gliosis	(Gonzalez Deniselle et al. 1996)
Stem cell therapy	Human cord blood mononuclear cells (injected in the brain ventricle)	Beneficial, improved motor performance, but did not reach the spinal cord	(Bigini et al. 2011)
	Human skeletal muscle-derived stem cells (injected in the brain ventricle)	Beneficial, improved motor performance, but did not reach the spinal cord	(Canzi et al. 2012)
	Human amniotic fluid cells (injected in the brain ventricle)	No or mild beneficial effect on motor performance no effect on survival	(Bigini et al. 2012)

To better describe the different trials approached in the wobbler mouse, treatments will be clustered by associating them with the different classes of drugs.

### Trophic factors and anti-inflammatory agents

In the last 20 years, several pharmacological treatments were performed with neurotrophic molecules, cytokines, soluble receptors and/or antiinflammatory compounds in wobbler mice. Mitsumoto and colleagues demonstrated that co-treatment with the ciliary neurotrophic factor (CNTF) and the brain-derived neurotrophic factor (BDNF) hugely delayed motor impairment by directly acting on the survival of the cervical spinal cord motor neurons, and that even the administration of CNTF or BDNF alone was able to slow down symptom progression, even though to at a lesser extent (Mitsumoto et al. 1994). Further treatments demonstrated that exogenous BDNF had a beneficial effect by partially inhibiting the increase of nitric oxide synthase (NOS) activity in spinal cord of wobbler mice (Tsuzaka et al. 2001). These results suggest that the BDNF is an important factor in reducing nitric oxide (NO)-mediated motor neuron degeneration. Similarly to human patients, both clinical and pharmacokinetic properties of recombinant BDNF with N-terminal methionine (as used in preclinical and clinical tests) and without (as the endogenous BDNF) were compared in early symptomatic wobbler mice. Both BDNF versions improved grip strength and running time of symptomatic mice while plasma levels of BDNF did not differ between the two groups. Met-free BDNF exerted similar effects compared to met-BDNF in wobbler mice (Ishiyama et al. 2002). The effect of recombinant human insulin-like growth factor-I (IGF-I) has been tested in a series of clinical trials in wobbler mice. The first study showed an increase of muscular strength (about 40 %) in IGF-I treated wobbler mice versus placebo-treated mice. In addition, IGF-I treated mice showed a marked weight increase from 3 to 6 weeks of treatment compared to controls. However, neither muscle activity nor the rate of motor neuron loss was modified by the treatment (Ikeda et al. 1995a). The co-treatment with IGF-I and glycosaminoglycans (GAGs) markedly improved the nerve regrowth and muscle reinnervation and consequently delayed the clinical progression in wobbler mice (Gorio et al. 1998; Vergani et al. 1999). A similar effect was observed even with reduced dosage of IGF-I (Gorio et al. 2002). Cardiotrophin-1 (CT-1), a powerful cardiac hypertrophic factor, significantly prevented the decline of motor performance of wobbler mice and revealed an increase in the number of motor neurons accompanied by a protection of large myelinated motor axons. The authors hypothesized that CT-1 might also provide a myotrophic effect besides a neurotrophic action (Mitsumoto et al. 2001). Although a beneficial role of neurotrophic factors has been suggested also by other studies with mouse models (Ikeda et al. 2000b); (Iwasaki et al. 2002), their effects in ALS patients were very poor and somehow controversial (Borasio et al. 1998; Beck et al. 2005). The lack of translational value of these studies might be due to the starting point of the treatment; patients are treated at a later stage of the disease, or might be attributed to differences in terms of bio-availability and/or toxicity of these compounds.

The treatment with the cytokine leukaemia inhibitory factor (LIF) (Kurek et al. 1998) that has both myotrophic and anti-inflammatory potentials, has been shown to slow down the progression of fore-limb atrophy accompanied by a significant preservation of foreleg muscle strength compared to vehicle-treated wobbler mice (Ikeda et al. 1995a). The inhibition of inflammatory pathways has been shown to partially counteract the clinical progression in wobbler mice. The co-treatment with interleukin 6 (IL-6) and soluble IL-6 receptor (sIL-6R) improved symptomatic and neuropathological progression in wobbler mice (Ikeda et al. 1996). It has been suggested that IL-6/sIL-6R complex might act on motor neurons through activation and homodimerization of gp130 (Ikeda et al. 1996). In a similar way, the systemic administration of a recombinant human TNF-binding protein-1 (rhTBP-1) delayed both symptom progression and motor neuron loss in the wobbler mouse by selectively inhibiting the expression of TNF-alpha and TNFR1 and phosphorylation of JNK and p38MAPK in affected motor neurons (Bigini et al. 2008). The link between the inflammatory reaction and motor neuron damage in wobbler mice has been recently confirmed by a treatment with a cyanobacterium-derived toll-like receptor 4 (TLR4), VB3323 (De Paola et al. 2012). In agreement with the results emerging from in vitro studies, in which VB3323 reduced motor neuron loss and microglial response after lipopolysaccharide (LPS) stimulation, a significant decrease in microglial activation and morphological alterations of motor neurons, associated with a significant improvement of motor performance was detected in VB3323-treated mice, as compared to both vehicle-treated and riluzole-treated wobbler individuals (Fumagalli et al. 2006).

In recent years, it has widely been demonstrated that the neuroprotective effect of erythropoietin and its derivates is related to both, trophic-antiapoptotic activity and anti-inflammatory action (Ghezzi and Brines 2004). In agreement with this, the beneficial effect of chronic treatment with non-hematopoietic erythropoietin derivatives has been demonstrated in wobbler mice. This was associated with improvement of motor function, decreased motor neuron loss and milder astrocyte- and microglia activation in the cervical spinal cord of wobbler mice, without any measurable effect on hematocrit values (Mennini et al. 2006).

### Antiglutamatergic agents

The role of glutamate-induced excitotoxicity has been greatly debated in ALS pathogenesis, to test whether motor neuronal death in wobbler mice is actually associated with an overstimulation of NMDA receptors, a treatment with the NMDA receptor antagonist, MK801, was carried out in wobbler mice starting just after birth or in the early symptomatic phase. In the group of animals treated from 4 days after birth onward, MK801 was highly toxic, whereas the group treated after 3–4 weeks of age well tolerated even higher dosage without side effects, such as lethargy or ataxia. However, no significant difference in the body weight and in the progression of symptoms was reported by comparing in MK801-treated and saline-treated wobbler mice (Krieger et al. 1992). To evaluate if the effectiveness of riluzole (Ishiyama et al. 2004) was exclusively associated with the antiglutamatergic hyperpolarizing effect of this compound (Benavides et al. 1985), two parallel studies were carried out in wobbler mice. These were aimed to compare the effects of riluzole and the AMPA receptor antagonist, RPR119990, on the clinical and neuropathological progression, characteristic for the wobbler mouse. As previously mentioned, the chronic treatment with riluzole improved motor behaviour, prevented biceps atrophy and reduced the decay of moto neuron numbers. RPR119990 in contrast was completely ineffective (Fumagalli et al. 2006). These results, together with the evidence of unchanged levels for the AMPA subunit GluR2 and NMDAr in the spinal cord and in motor neurons of symptomatic wobbler mice (Bigini et al. 2006), suggest that the protective effect of riluzole in wobbler mice is independent of its antiglutamatergic activity.

### Mitochondrial suppliers and antioxidants agents

The implication of mitochondrial deficits in the evolution of wobbler MND has been highlighted by different and independent treatments in which energetic substrates could attenuate the clinical progression of wobbler mice. The treatment with creatine monohydrate showed delayed denervation, reduced muscle atrophy and motor neuron loss and led to a significant improvement of behavioural performance (Ikeda et al. 2000a). The link between the efficiency of mitochondrial activity and the beneficial effect on wobbler mice was confirmed by the evidence that early symptomatic wobbler mice exposed to hyperbaric oxygen therapy (HBOT) showed both delayed onset of symptoms and significantly improved motor performance (Dave et al. 2003b). Based on these results, the effect of acetyl-l-carnitine (ALCAR) was tested in wobbler mice by two different approaches. The rationale of these studies was to connect the well-known effect of ALCAR on mitochondrial activity (Bertamini et al. 2002) with the neuroprotective and neurotrophic activity seen in cultured primary motor neurons from rat embryos (Bigini et al. 2002). Unexpectedly, neither the systemic treatment (intraperitoneal injection) and the application with minipumps had any beneficial effect, nor on the clinical symptoms and neuropathological effects in early symptomatic wobbler mice (Bigini et al. 2002); (Beghi et al. 2011).

Since an impaired electron transport chain is often accompanied by an overproduction of reactive oxygen species, the possible beneficial effects of scavengers were evaluated in wobbler mice. The treatment with the free radical scavenger, OPC-14117, improved motor activity and reduced forelimb weakness in a dose-dependent manner. The mechanism appears to be associated with a decreased formation of lipid peroxides, which might diminish motor neuron death (Abe et al. 1997). A similar effect was observed when treating wobbler mice with the antioxidant lecithinized superoxide dismutase. In particular, the latter compound prevented denervation, muscle atrophy and delayed degeneration of spinal motor neurons in wobbler mice (Ikeda et al. 1995b). A further study, conducted in early symptomatic wobbler mice with a nonselective NOS inhibitor, l-NAME, sustained grip strength and attenuated the loss of motor neurons and disease progression (Ikeda et al. 1998).

### Steroid hormones

Since the early 1990s an important contribution to the understanding of the potential role of progesterone, steroid receptors and glucocorticoids in enhancing degenerative processes or in protecting motor neurons in wobbler mice has been provided by De Nicola and colleagues. In particular, a significant improvement of muscle function was observed in wobbler mice with a single progesterone pellet implanted. The beneficial effect on motor performance was associated with a marked reduction of cytoplasmic vacuolization in the degenerating motor neurons, a decrease of growth-associated protein (GAP-43) mRNA expression and mitigation of reactive gliosis (Gonzalez Deniselle et al. 2002; Deniselle et al. 2012). Another effect of progesterone treatment was a reduced expression of nicotinamide adenine dinucleotide phosphate-diaphorase (NADPHD) and NOS in spinal cords of symptomatic wobbler mice (Gonzalez Deniselle et al. 2005). A similar neuroprotective function has been suggested for the 21-aminosteroid, U-74389F, leading to increased muscle trophism, reduced oxidative stress and decreased glial response (Gonzalez Deniselle et al. 1996, 1999).

One of the most interesting results emerging from the treatment with riluzole in wobbler mice was the selective increase of BDNF immunoreactivity in cervical motor neurons (Fumagalli et al. 2006). The evidence that the treatment with progesterone could increase the mRNA levels of BDNF in spinal cords of early and late symptomatic wobbler mice (Gonzalez Deniselle et al. 2007) confirmed the therapeutic value of BDNF in MND and supports the hypothesis that the beneficial effect of riluzole is not exclusively dependent on its antiglutamatergic activity.

### Stem cell therapy

Many further trials with different types of pharmacological compounds, such as gangliosides, plasminogen activators, anticholesteremic agents have been reported to be effective in wobbler mice (Blondet et al. 1992; Bose et al. 1999; Lisovoski et al. 1997; Iwamoto et al. 2009). However, despite these encouraging results, the clinical trials of some of these compounds in ALS patients had no significant effect. Thus, new innovative strategies are required and have been developed to the preclinical level. Among those, stem cell (SC) therapy is considered one of the most promising approaches for the treatment of ALS and other neurodegenerative disorders. In recent years, the wobbler mouse has been successfully employed as an ALS model to test the efficacy of different types of stem cells and to determine the interaction between transplanted cells and host tissues (biodistribution, half-life, clearance, accumulation).

Among the cell therapy approaches, the systemic injection of human cord blood mononuclear cells (HuCB-MNCs) has proven to reproducibly increase, the life span of SOD1G93A mice in a dose-dependent manner (Garbuzova-Davis et al. 2008). In order to verify their potential in other models of motor neuron degeneration and by other routes of administration, HuCB-MNCs, labelled with the nuclear dye

Hoechst 33258, were directly implanted in the lateral brain ventricles of early symptomatic wobbler mice. HuCB-MNCs transplantation significantly ameliorated the symptoms and reduced the rate of motor neuron death and gliosis (Bigini et al. 2011). The lack of Hoechst 33258-positive cells at the spinal cord level suggested that the beneficial role of transplanted cells is not due to the cell replacement, but is rather associated with the production and release of circulating protective factors. The potential of fetal cell transplantation has also been demonstrated. “Human skeletal muscle-derived stem cells (SkmSCs)” showed a positive effect, after implantation in the brain lateral ventricles of wobbler mice. Like HuCB-MNCs, transplanted SkmSCs did not reach the ventral horns of cervical spinal cords, but magnetic resonance imaging (MRI) showed that some clusters of cells migrated in the spinal parenchyma and the signal was maintained up to 18 weeks after transplantation (Canzi et al. 2012). The evidence of a marked expression of specific human anti-inflammatory cytokines in the brain of transplanted wobbler mice seems to confirm the hypothesis that a bystander effect of soluble factors, released by SkmSCs, even far from the affected areas, might play a neuroprotective role.

Finally, the combination of super paramagnetic iron oxide nanoparticles (SPIOn) and Hoechst 33258 was used to track human amniotic fluid cells (hAFCs) after transplantation in the lateral ventricles of wobbler- and healthy mice. In vivo and ex vivo approaches have shown that hAFCs rapidly spread to the whole ventricular system, but did not migrate into the brain parenchyma independently from the health status of the individual mouse. The transplanted cells survive for a long time in the ventricles of both wobbler and healthy mice, but had no influence on the survival of mice and did not cause inflammation or pleocytosis (Bigini et al. 2012).

## Conclusions

The phenotype of the wobbler mouse closely resembles human ALS regarding motor neuron degeneration, muscle atrophy, reactive gliosis as well as the cellular effects like ubiquitinated protein aggregation, oxidative stress, cortical hyperexcitability and cellular transport defects. Thus, the wobbler mouse models the complexity of ALS pathology with a number of interdependent cellular defects, the precise contribution of which is currently unclear. The ALS-like wobbler phenotype is caused by a point mutation in Vps54, a component of the GARP vesicle-tethering complex. The point mutation leads to a decreased stability of the GARP complex and thereby to impairments in the retrograde vesicle transport from endosomes to the TGN. These impairments consequently lead to missorting of proteins, accumulation of abnormally enlarged APP-positive endosomal compartments as it is also seen in a subset of human ALS patients. Even though there is no gap-less chain of evidence, it appears plausible that the impairment of the retrograde vesicle traffic leads to disturbance of several cellular processes such axonal protein- and organelle transport and the final consequences could be protein aggregation and mitochondrial dysfunction. The therapeutic interventions tested on wobbler mice have also contributed to the understanding of the complex pathology of ALS. The beneficial effects seen for anti-inflammatory- and antioxidant agents indicate that neuroinflammation and oxidative stress also plays a critical role in the wobbler motor neuron degeneration, while the poor effect of antiglutamatergic compounds might argue that glutamate-associated excitotoxicity plays a minor role. Our recent finding of hyperexcitability due to a reduced number of GABAergic interneurons in wobbler CNS, even prior to the disease onset appears to be more difficult to explain. However, similar cortical hyperexcitability has been reported in human ALS cases, also already prior to the onset of disease symptoms and has been attributed to the degeneration of inhibitory circuits (Vucic et al. 2009). Loss or degeneration of inhibitory neurons appears to be an early event in the pathology and thus might have a diagnostic and a therapeutic impact, both of which could be addressed using the wobbler mouse. Thus, the wobbler mouse is, even 57 years after its first description, still a valuable animal model for human MNDs such as ALS, in order to unravel the disease mechanism and in order to develop and test new diagnostic and therapeutic approaches.
